# Impact of Dietary Supplements on Clinical Outcomes and Quality of Life in Patients with Breast Cancer: A Systematic Review

**DOI:** 10.3390/nu17060981

**Published:** 2025-03-11

**Authors:** Luca Scafuri, Carlo Buonerba, Oriana Strianese, Evandro de Azambuja, Michela Palleschi, Vittorio Riccio, Vincenzo Marotta, Concetta Scocca, Giovanni Riccio, Carla Errico, Grazia Arpino, Giuseppe Di Lorenzo

**Affiliations:** 1 Oncology Unit, “Andrea Tortora” Hospital, ASL Salerno, 84016 Pagani, Italy; lucaluca@hotmail.it (L.S.); xt.strianeseo@aslsalerno.it (O.S.); g.dilorenzo@aslsalerno.it (G.D.L.); 2 Associazione O.R.A. ETS-Oncology Research Assistance, 84134 Salerno, Italy; 3 Institut Jules Bordet, l’Université Libre de Bruxelles, 1070 Brussels, Belgium; evandro.deazambuja@hubruxelles.be; 4 Medical Oncology, Breast & GYN Unit, IRCCS Istituto Romagnolo per lo Studio dei Tumori (IRST) “Dino Amadori”, 47014 Meldola, Italy; michela.palleschi@irst.emr.it; 5 Ospedale SS Maria della Pietà, 80026 Casoria, Italy; vittorioriccio1990@gmail.com; 6 UOC Clinic of Endocrinology and Diabetology, AOU San Giovanni di Dio and Ruggi d’Aragona, 84131 Salerno, Italy; vinc.endo@libero.it; 7 Lincolnshire Pain Service Connect Health, Newcastle Upon Tyne NE12 8EU, UK; concettascocca@gmail.com; 8 Department of Medicine, University “Luigi Vanvitelli” of Naples, 80138 Naples, Italy; ricciogiovanni1992@gmail.com; 9 A.O.U. Vanvitelli, Internal Medicine—San Paolo Hospital Campus (Fuorigrotta), 80142 Naples, Italy; carlaerrico21@gmail.com; 10 Department of Clinical Medicine and Surgery, University of Naples Federico II, 80133 Naples, Italy; grazia.arpino@unina.it; 11 Department of Medicine, UniCamillus-Saint Camillus International University of Health Sciences, 00131 Rome, Italy

**Keywords:** supplement, quality of life, well-defined substances, vitamin, herbal extract

## Abstract

**Background:** This systematic review aimed to evaluate the efficacy and safety of dietary supplements in breast cancer patients, focusing on their impact on clinical outcomes, treatment-related side effects, and therapy adherence. **Methods:** Only RCTs investigating the effects of various orally administered supplements in adult breast cancer patients were included. Well-defined substances like vitamins, minerals, antioxidants, and specific herbal extracts were explored. The review excluded studies solely based on dietary interventions or non-supplemental approaches. The primary outcome assessed was quality of life. Secondary outcomes included disease-free survival, overall survival, tumor response, and biomarkers indicative of disease progression. **Results:** A total of 45 randomized controlled trials (RCTs) were included in this systematic review. Overall, supplementation was not associated with serious adverse events in the included trials. Vitamin D supplementation showed promise in some studies, with potential immunomodulatory and antioxidant effects, particularly when combined with other interventions. Omega-3 fatty acids and beta-glucan demonstrated potential in alleviating certain symptoms and improving quality of life. Studies on amino acids like acetyl-L-carnitine and L-arginine also yielded mixed results. Beta-glucan exhibited potential for immune-enhancing effects, while melatonin and creatine showed limited or no benefit for fatigue or muscle strength. Herbal extracts, including silymarin, curcumin, and EGCG, had varied effects. Curcumin studies presented mixed results. Silymarin showed potential for hepatoprotective effects. **Conclusions:** These findings highlight the potential of specific dietary supplements to improve various aspects of breast cancer care. However, the evidence is mixed across supplement types, and further research is needed to determine the most effective and safe approaches.

## 1. Introduction

Breast cancer remains one of the most prevalent malignancies worldwide, affecting millions of women each year and representing a significant global health challenge [1]. Advances in standard treatments, including surgery, chemotherapy, radiotherapy, and targeted therapies, have substantially improved survival rates and clinical outcomes [2]. Despite these successes, these interventions often result in debilitating side effects, such as fatigue, nausea, immune suppression, and long-term health complications, which can severely impact patients’ quality of life [3]. Addressing these challenges has led to growing interest in complementary approaches, particularly dietary supplements, as potential adjuncts to conventional treatments to improve therapeutic outcomes and manage side effects [4,5]. Dietary supplements, including vitamins, minerals, antioxidants, and herbal extracts, are increasingly used by patients with breast cancer to support the side effects of their oncological treatments. Unlike supplements used for prevention, this review focuses on their role in patients already diagnosed with breast cancer, addressing their potential to manage treatment-related side effects, enhance adherence to therapy, and improve clinical outcomes [6]. Randomized controlled trials (RCTs) have explored the impact of these supplements, with some studies reporting promising benefits, such as improved quality of life, reduced adverse effects, and extended survival. For instance, a large RCT investigating Vitamin D supplementation demonstrated reduced recurrence rates and improved overall survival in specific subgroups of patients with breast cancer, highlighting the potential of targeted supplement use [7].

The potential benefits of supplements in breast cancer management extend across several domains. Certain supplements, such as antioxidants, may mitigate oxidative stress and reduce damage associated with chemotherapy and radiotherapy [8]. For example, Vitamin C has been hypothesized to alleviate oxidative damage, while omega-3 fatty acids have been recognized for their anti-inflammatory properties, which may help address inflammation and other risk factors linked to disease progression [9]. Additionally, supplements may improve therapy compliance by alleviating side effects that could otherwise lead to treatment discontinuation or dose modifications [4,5]. Other supplements, such as curcumin or green tea polyphenols, have been explored for their roles in influencing biomarkers of disease progression, including inflammatory cytokines and tumor markers, suggesting additional therapeutic benefits [3,10].

While several systematic reviews have investigated the role of dietary supplements in breast cancer, significant limitations within the existing literature necessitate this current review. Some reviews, such as those focusing on isoflavones [11,12], concentrate on a narrow range of compounds, primarily derived from soy, and their impact on hormone-dependent cancers or risk factors. This leaves a gap in understanding the effects of other widely used supplements, like vitamins, minerals, and various herbal extracts, on broader clinical outcomes. Other reviews, like the one by Becerra-Tomás et al. [13], examine post-diagnosis dietary factors and supplement use broadly, but blend dietary interventions with supplement use, making it difficult to isolate the specific effects of supplements themselves. The ASCO-Society for Integrative Oncology Guideline Update [14] on fatigue management, while valuable, is a clinical guideline and focuses primarily on one symptom, rather than a comprehensive review of supplement efficacy across a range of outcomes. Reviews such as those addressing probiotics [15], aromatase inhibitor-induced symptoms [16], or hot flash management [17] are very narrowly focused on specific supplement types or symptoms. Furthermore, many of these reviews analyze a mixture of study designs, potentially including observational studies, diluting the strength of causal inference. In contrast, this present review addresses these shortcomings by exclusively focusing on randomized controlled trials (RCTs) of well-defined, orally administered supplements (excluding broad dietary interventions), and by assessing their impact on a comprehensive set of outcomes, including quality of life, treatment-related side effects, and disease progression, thereby providing a more rigorous and clinically relevant synthesis of the evidence.

Given the increasing interest in dietary supplements among breast cancer patients, this systematic review aims to evaluate the efficacy and safety of dietary supplements in patients with breast cancer, focusing on their role in improving clinical outcomes, managing treatment-related side effects, and enhancing therapy adherence. By focusing on well-defined substances, such as vitamins and minerals, alongside herbal extracts, this review seeks to delineate their roles in breast cancer care and evaluate their efficacy in improving key outcomes, including survival, quality of life, and the management of treatment-related side effects. The ultimate goal is to identify promising supplements, assess the strength of the evidence supporting their use, and provide insights into their risks and benefits for both clinical practice and future research. Following the PRISMA guidelines, this systematic review ensures a rigorous and transparent methodology in data collection and analysis, contributing to a deeper understanding of the potential integration of dietary supplements into comprehensive breast cancer management strategies [18].

## 2. Methods

This systematic review included randomized controlled trials (RCTs) investigating the effects of orally administered supplements in adults (≥18 years old) with a histologically confirmed diagnosis of breast cancer, encompassing all stages (I-IV) and subtypes. Eligible interventions included single, well-defined substances such as vitamins, minerals, antioxidants, probiotics, prebiotics, and fish oil, as well as herbal extracts. Studies that evaluated whole foods, specific diets, or other dietary interventions without a distinct supplement component, as well as those focused on non-supplement interventions, were excluded. The primary outcome was quality of life. Secondary outcomes included disease-free survival, overall survival, treatment-related adverse events, tumor response, and biomarkers of disease progression.

To comprehensively identify relevant research on the use of oral dietary supplements in adult breast cancer patients, we conducted a systematic search of the literature. This search encompassed major electronic databases, including PubMed/MEDLINE, Embase, the Cochrane Central Register of Controlled Trials (CENTRAL), and Web of Science. All database searches were last updated on 1 October 2024. Our search strategy combined Medical Subject Headings (MeSH) terms and free-text keywords related to “breast neoplasms” (and related terms) AND “dietary supplements” (and related terms). In addition, we specifically searched for substances of interest, including “vitamin D”, “omega-3 fatty acids”, “curcumin”, “beta glucan”, “silymarin”, and “EGCG”, along with their respective synonyms, related compounds, and relevant MeSH terms where applicable.

To ensure a thorough search, we also manually examined clinical trial registries (e.g., ClinicalTrials.gov), relevant conference proceedings, and the reference lists of all included studies to identify any additional publications that might have been missed by the electronic searches. No restrictions were placed on language or publication date to maximize the breadth of our search. After initial screening, which was performed independently by two authors (C.B. and L.S.), studies were included in our review if they met the following criteria: the study population consisted of adult patients diagnosed with breast cancer; the intervention involved the oral administration of dietary supplements, encompassing vitamins, minerals, antioxidants, or specific herbal extracts; and the study design was a randomized controlled trial (RCT). We prioritized studies that reported on quality of life as the primary outcome. Secondary outcomes of interest included disease-free survival, overall survival, the incidence and severity of treatment-related adverse events, tumor response rates, and changes in biomarkers indicative of disease progression. Studies were excluded if they focused solely on dietary interventions (without specific supplementation) or non-supplemental approaches. This rigorous selection process ensured that only high-quality, relevant evidence was included in our review. Trials were included regardless of the risk of bias.

The risk of bias in included RCTs was assessed using the Cochrane Risk of Bias tool (RoB 2) [19]. This tool evaluates potential bias across domains, including the randomization process, deviations from intended interventions, missing outcome data, outcome measurement, and selective reporting. Each study was independently assessed by two reviewers (L.S. and C.B.), with disagreements resolved through discussion or consultation with a third reviewer (G.A.), ensuring consistency in evaluating study quality.

**Figure 1:**
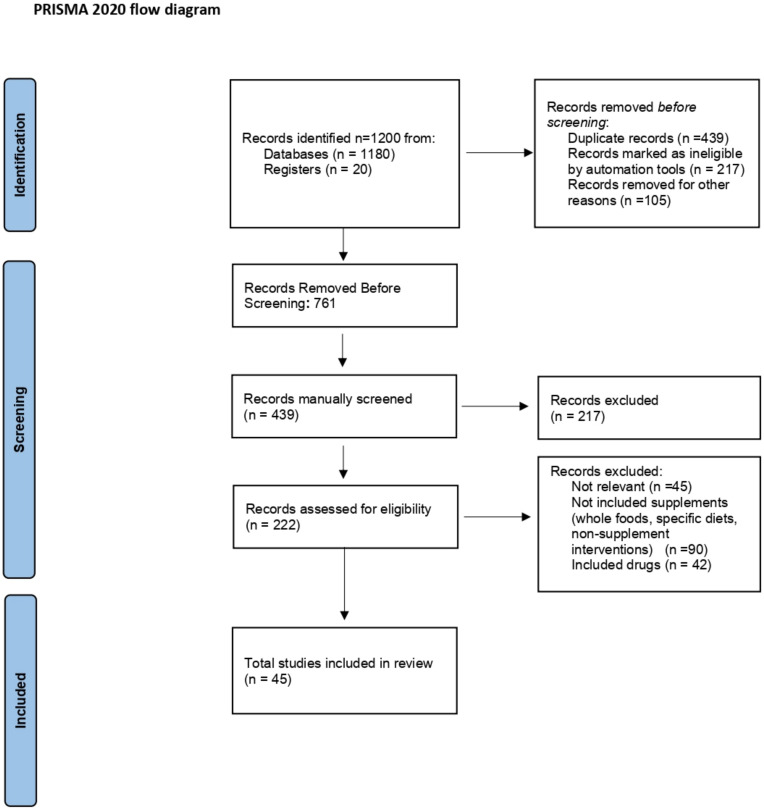
PRISMA 2020 flow diagram.

We conducted a narrative synthesis with summary statistics, categorizing studies by supplement type. Data from each trial—including participant characteristics, interventions, outcomes, and risk of bias assessments—were systematically compiled into summary tables. This approach enabled a comprehensive narrative analysis within each supplement category, emphasizing the direction and magnitude of effects, consistency across trials, and potential sources of heterogeneity. To enhance clarity and transparency in the study selection process, we include a PRISMA flow diagram that visually represents the number of records identified, screened, included, and excluded, along with reasons for exclusion at each stage. (Figure 1).

The flow diagram illustrates the study selection process for the systematic review. A total of 1200 records were identified from databases and registers, with 20 records specifically from registers. After removing duplicates, screening records, and assessing eligibility, 45 studies were included in the final review. The diagram follows the PRISMA 2020 guidelines, detailing the identification, screening, eligibility, and inclusion stages.

## 3. Results

### 3.1. Included Studies

#### Search Results and Study Selection

A systematic search of the literature yielded 1200 records, of which 439 underwent manual screening. Exclusion criteria included irrelevance to the research question (*n* = 45), focus on non-supplement dietary interventions (*n* = 90), or use of drugs instead of supplements (*n* = 42). This resulted in 217 records being excluded and 222 assessed for eligibility. High inter-rater agreement was observed during screening (Cohen’s kappa = 0.91).

Eligibility assessment, based on pre-defined criteria (adult breast cancer patients, oral dietary supplement intervention, randomized controlled trial design, and reporting on quality of life outcomes), resulted in the exclusion of 177 articles. Ultimately, 45 randomized controlled trials met all inclusion criteria and were included in the final systematic review. These 45 studies investigated the effects of a range of orally administered dietary supplements in adults with breast cancer. A total of 45 randomized controlled trials (RCTs) were included in this systematic review, investigating the effects of orally administered supplements in adults (≥18 years old) with a histologically confirmed diagnosis of breast cancer. These studies involved a diverse sample of patients across all stages (I-IV) and subtypes of breast cancer, including those undergoing chemotherapy or in the post-treatment phase. Eligible interventions comprised well-defined substances such as vitamins (e.g., Vitamin D), minerals, antioxidants, omega-3 fatty acids, creatine, and specific herbal extracts, including silymarin, curcumin, and grape seed extract. Studies focusing solely on dietary interventions (e.g., whole foods, specific diets) or non-supplement interventions (e.g., probiotics, prebiotics) were excluded to ensure that the review focused solely on the impact of supplements. The primary outcome assessed in this systematic review was quality of life. Secondary outcomes included disease-free survival, overall survival, treatment-related adverse events, tumor response, and biomarkers. Most of the studies employed placebo-controlled designs, with control groups receiving either a placebo or standard care, enhancing the validity of the findings. Overall, supplementation was not associated with serious adverse events in the included trials. The following is a detailed narrative analysis of the results obtained in the included trials (Supplementary Materials, Tables S1 and S2).

### 3.2. Synthesis of Results

#### 3.2.1. Vitamins and Minerals

Vitamin D3, or cholecalciferol, is a fat-soluble vitamin essential for calcium and phosphorus homeostasis, bone health, and immune system regulation. The studies on Vitamin D and mineral supplementation in breast cancer patients yielded mixed results. Some studies indicated potential benefits of Vitamin D on inflammation, but the effects on tumor-related outcomes were inconclusive. Tirgar et al. (2024) [1] conducted a pilot RCT involving 88 breast cancer patients to assess the combined effects of vitamin D and synbiotics on inflammation and treatment response. The study found a significant increase in serum vitamin D levels in the intervention groups, with stabilized IL-10 levels and an improved anti-inflammatory index. However, no significant differences were observed in tumor growth markers, residual tumors, or metastasis.

Similarly, Mohseni et al. (2019) [20] found that Vitamin D3 supplementation (*n* = 56) significantly increased serum 25(OH)D levels (from 28 to 39 ng/mL, *p* = 0.004) and total antioxidant capacity (TAC, *p* ≤ 0.04), with responses influenced by VDR genotypes. However, inflammatory markers TNF-α and TGF-β1 remained unchanged. Naderi et al. (2022) [21] highlighted a synergistic anti-inflammatory effect of high-dose Vitamin D combined with yoga in breast cancer survivors, showing increased IL-10 levels and enhanced IL-10/TNF-α ratios. Dastmardi et al. (2024) [22] evaluated Vitamin D’s protective role in ovarian reserve preservation in 33 breast cancer patients undergoing chemotherapy. While AMH levels decreased in both groups, the Vitamin D group showed a slight but non-significant trend toward ovarian protection (*p* = 0.054). Amenorrhea rates were comparable between groups six months post-chemotherapy. These reported results confirmed a non-significant trend toward better ovarian reserve preservation during chemotherapy (*p* = 0.054) with Vitamin D supplementation (*n* = 33). McGuinness et al. (2024) [7] investigated Vitamin D’s impact on breast cancer risk using a convolutional neural network (CNN)-based mammographic evaluation. After 12 months, there were no significant differences in mammographic density or CNN-based breast cancer risk scores between Vitamin D and placebo groups, suggesting that Vitamin D does not substantially alter imaging-based risk indicators. Other studies, including those published by Shahvegharasl (2020) [23] and Niravath et al. (2019) [24], showed that Vitamin D supplementation improved serum Vitamin D levels but was not associated with any significant effects on angiogenic biomarkers or aromatase inhibitor-induced arthralgia, respectively. Shahvegharasl et al. (2020) [23] assessed Vitamin D supplementation in 44 breast cancer patients receiving tamoxifen. While patients in the intervention group had significantly higher serum Vitamin D levels (*p* < 0.05), no significant changes were observed in angiogenic biomarkers. Niravath et al. (2019) [24] investigated high-dose versus standard-dose Vitamin D3 supplementation in 93 breast cancer patients. Despite a significant increase in Vitamin D levels (*p* < 0.0001), there was no correlation between Vitamin D levels and the development of aromatase inhibitor-induced arthralgia, leading to the study’s early termination.

Overall, while Vitamin D supplementation appears to enhance anti-inflammatory and antioxidant outcomes, its impact on tumor markers and clinical cancer outcomes remains inconclusive, requiring further investigation in larger, diverse populations.

#### 3.2.2. Omega-3 Fatty Acids

Docosahexaenoic acid (DHA) and eicosapentaenoic acid (EPA) are long-chain omega-3 fatty acids primarily derived from marine sources like fish oil, known for their anti-inflammatory properties and beneficial roles in supporting bone health. Research suggests that DHA and EPA supplementation may offer benefits for breast cancer patients, particularly in reducing treatment-related side effects. Given the potential bone-protective effects of eicosapentaenoic acid (EPA) and docosahexaenoic acid (DHA) on cellular processes related to bone turnover, a randomized, double-blind, placebo-controlled pilot study was conducted to evaluate whether supplementation with 4 g of EPA and DHA daily for three months could mitigate bone turnover in this population [25]. The study included 38 postmenopausal women undergoing AI therapy, with analyses performed at baseline and after three months to assess serum fatty acid composition, bone turnover markers, and inflammatory markers. The results indicated that supplementation with EPA and DHA significantly increased serum concentrations of total and long-chain (LC) omega-3 polyunsaturated fatty acids (PUFAs) while simultaneously reducing levels of arachidonic acid, total LC omega-6 PUFAs, and the LC omega-6:omega-3 PUFA ratio, relative to placebo (*p* < 0.05). Additionally, bone resorption was inhibited among participants who responded to fish oil supplementation, further supporting its potential role in counteracting AI-induced bone turnover (*p* < 0.05).

Omega-3 supplementation demonstrated various benefits in breast cancer patients, particularly in improving symptoms and managing side effects of treatment. De la Rosa Oliva et al. (2019) [26] found omega-3 helpful for reducing chemotherapy-induced xerostomia and improving overall quality of life. In a double-blinded, placebo-controlled trial involving 53 women with locally advanced breast cancer, participants receiving 2.4 g/day of omega-3 fatty acids (EPA 1.6 g, DHA 0.8 g) showed significant improvement in xerostomia (dry mouth) compared to the placebo group (*p* = 0.032). Darwito et al. (2019) [27] reported improved progression-free survival (28.5 ± 3.3 weeks vs. 23.7 ± 3.6 weeks, *p* = 0.044) and overall survival (30.9 ± 3.7 weeks vs. 25.9 ± 3.6 weeks, *p* = 0.048) in 48 patients supplemented with 1 g/day of omega-3 during chemotherapy. Significant reductions in Ki-67 expression (*p* = 0.032) and VEGF levels (*p* = 0.041) were also noted, indicating anti-tumor activity. Hutchins-Wiese et al. (2014) [25] assessed omega-3 supplementation in 38 postmenopausal breast cancer survivors on aromatase inhibitors. The study found significant increases in EPA, DHA, and total omega-3 levels, leading to reduced bone resorption (*p* < 0.05), suggesting potential benefits in preventing AI-induced bone loss. The study demonstrated a reduction in bone resorption rates with EPA/DHA supplementation (4 g/day) in 38 postmenopausal breast cancer survivors on aromatase inhibitors (*p* < 0.05), suggesting potential protection against treatment-induced bone loss. Similarly, Ghoreishi et al. (2012) [28] found a significantly lower risk of peripheral neuropathy (PN) in 57 patients receiving omega-3 during paclitaxel therapy (OR = 0.3, *p* = 0.029), with a smaller decline in nerve conduction compared to placebo (*p* = 0.015). Less significant findings include a pilot trial conducted by Lustberg et al. (2018) [29] with 44 women randomized to receive omega-3 fatty acids or placebo for 24 weeks. While no significant differences in pain severity or interference were observed between groups, the placebo group experienced a significant decline in quality of life (QOL) by week 12, while the omega-3 group did not. Overall, omega-3 supplementation shows promise in managing chemotherapy-induced symptoms, improving quality of life, and mitigating treatment-related side effects, though further research is needed to confirm these findings and explore long-term outcomes.

Acetyl-L-carnitine is a modified amino acid that facilitates the transport of fatty acids into mitochondria, where they are used for energy production. Clinical studies have examined its role in managing chemotherapy-induced peripheral neuropathy (CIPN), a major quality-of-life concern in breast cancer patients and also a painful side effect for many cancer patients, making it a versatile supplement in oncology support [30]. L-arginine is a semi-essential amino acid involved in protein synthesis, immune response, and wound healing. Some studies have suggested that L-Arginine supplementation may help maintain lean body mass and improve fatigue levels, thus positively affecting quality of life in cancer patients. However, its role in breast cancer remains controversial due to its ability to promote nitric oxide (NO) production, which could support tumor growth in certain cases. Patient-specific evaluations are necessary to determine whether L-Arginine supplementation is beneficial or poses risks in breast cancer treatment [31].

Randomized controlled trials (RCTs) on amino acids showed mixed findings. In the SWOG S0715 trial involving 437 patients, 409 completed assessments for taxane-induced neuropathy. Hershman DL et al. (2018) [30] found that acetyl-L-carnitine (ALC) significantly worsened neuropathy symptoms compared to placebo, with average FACT-NTX scores decreasing over time (*p* < 0.001). At 104 weeks, 39.5% of ALC patients experienced clinically significant worsening of symptoms (*p* = 0.04). Additionally, higher weight was associated with an increased risk of worsening neuropathy in the ALC group. Conversely, in a randomized, double-blind, placebo-controlled trial by Heys SD et al. (1998) [31] involving 96 patients with large primary breast cancers undergoing neo-adjuvant chemotherapy, L-arginine supplementation (30 g/day) showed no significant difference in overall clinical response rates between the L-arginine and placebo groups. However, for tumors smaller than 6 cm, histopathological responses were significantly better in the L-arginine group (88%) compared to the placebo group (52%) (*p* = 0.04), suggesting potential benefits for specific patient subsets.

#### 3.2.3. Other Supplements

Melatonin, a hormone produced by the pineal gland, regulates sleep–wake cycles and serves as a potent antioxidant. In breast cancer, studies have explored melatonin’s effects on hormone receptor-positive tumors, suggesting it may counter estrogen-driven growth. Given its wide-ranging effects and low toxicity, melatonin is frequently studied as an adjunctive therapy for improved quality of life. Beta glucan is a polysaccharide found in the cell walls of yeast, oats, and mushrooms, known for its immune-modulating properties. Clinical studies suggest that Beta-glucan can stimulate immune cells such as macrophages and neutrophils, and other immune cells, enhancing the body’s defense against pathogens and potentially cancer cells [32]. Creatine is an amino acid derivative stored in muscles and the brain, where it helps regenerate adenosine triphosphate (ATP), the main energy currency of cells. It is commonly associated with muscle strength and endurance, which can be beneficial for patients with cancer experiencing muscle wasting and fatigue [33]. By supporting energy production and reducing oxidative stress, creatine may offer adjunctive benefits, although further research is needed to clarify its role in cancer care.

Studies on melatonin, creatine, and beta glucan supplementation in patients with breast cancer yielded mixed results. Ostadrahimi et al. (2014) [32], in a randomized double-blind placebo-controlled trial involving 30 women with breast cancer undergoing chemotherapy, evaluated beta glucan supplementation (10 mg daily for 21 days) for its effects on white blood cell counts and serum levels of IL-4 and IL-12. Both groups experienced a decrease in white blood cell counts, but the reduction was less significant in the beta glucan group. Notably, serum IL-4 levels significantly increased (*p* = 0.001), and IL-12 levels also rose significantly (*p* = 0.03) in the beta glucan group, suggesting its potential as an immunomodulatory adjunct in breast cancer treatment. In a double-blind, placebo-controlled trial by Mukhopadhyay et al. (2024) [6] on melatonin supplementation for cancer-related fatigue in early stage breast cancer patients undergoing radiotherapy, changes in fatigue scores (FACIT and PROMIS) showed no statistically significant treatment effects (*p* > 0.05). The melatonin group reported slightly worse depression scores compared to the placebo group (*p* = 0.0389), but this difference was not clinically meaningful. Overall, the study indicated little to no benefit of melatonin in alleviating fatigue or improving well-being. For creatine, Parsowith et al. (2024) [33] examined the effects of short-term creatine supplementation on muscular performance in breast cancer survivors. Nineteen female participants (mean age 57.6 years) were randomized into groups, but no significant interaction between time and group was observed across various muscular performance metrics, including body mass, sit-to-stand power, and peak torque. However, both groups showed a significant improvement over time in 10 RM chest press and leg extension strength (*p* < 0.001 and *p* = 0.003, respectively), indicating an increase in muscular strength independent of creatine supplementation. Creatine did not significantly impact other performance outcomes. However, creatine supplementation did not provide a clear advantage over placebo in muscular performance metrics, including body mass and peak torque. Overall, beta glucan demonstrated significant immune benefits during chemotherapy, while melatonin and creatine showed limited or inconclusive effects in alleviating fatigue or enhancing muscular performance beyond general improvements seen in controls.

#### 3.2.4. Herbal Extracts

Herbal extracts play a crucial role in complementary and integrative medicine, with research exploring their applications in managing chronic diseases, including cancer, cardiovascular health, and metabolic disorders [34].

In nutritional supplements, herbal extracts support health conditions such as digestive issues, immune dysfunction, and stress. Key examples include the following:

Silymarin (Milk Thistle): Known for its hepatoprotective properties, silymarin protects liver cells from toxins, including chemotherapy drugs, and supports liver regeneration. It also has anticancer effects, inhibiting cancer cell proliferation, inducing apoptosis, and preventing metastasis. Silymarin modulates hormone receptors in breast cancer and alleviates chemotherapy-related side effects [35,36].

Two RCTs assessed silymarin’s hepatorenal protective effects in patients with breast cancer undergoing chemotherapy. Erfanian SS et al. (2024) [35] assessed the hepatorenal protective effects of silymarin in 100 patients with breast cancer undergoing chemotherapy. They found significant liver function improvements, particularly in alkaline phosphatase (ALP) and bilirubin levels, indicating potential hepatoprotective effects. However, no significant renal protection was observed. Silymarin was well-tolerated with minimal side effects. Overall, silymarin appears to offer liver protection in breast cancer patients receiving chemotherapy, though renal effects were inconclusive. 

Moezian GSA et al. (2022) [36] aimed to investigate the clinical efficacy of systemic administration of silymarin in the management of chemotherapy-induced hepatotoxicity in patients with non-metastatic breast cancer who received doxorubicin/cyclophosphamide-paclitaxel (AC-T) regimen. In this randomized, triple blind, placebo-controlled clinical trial, 30 patients who received AC-T who fulfilled the inclusion criteria were randomly allocated to silymarin (*n* = 15) or placebo (*n* = 15) groups to receive oral silymarin 140 mg three times a day or placebo tablets, respectively. Fatty liver severity was assessed by liver ultrasound imaging and also measurement of liver function tests before and after the intervention.

The study showed significant reductions in hepatic involvement based on ultrasonography in the silymarin group (*p* = 0.012). There was also a non-significant trend towards less severe liver involvement in the silymarin group compared to the placebo group (*p* = 0.083).

Curcumin (Turmeric) is widely studied for its anti-inflammatory, antioxidant, and anticancer properties [2,37].

Nano-Curcumin: An advanced curcumin formulation that improves absorption and therapeutic effects. It inhibits tumor growth, enhances treatment efficacy, and reduces side effects, improving patient adherence and quality of life. Nano-curcumin’s enhanced pharmacokinetics make it a promising adjunct to conventional therapies [38].

Curcumin supplementation studies in breast cancer patients showed varied results. Lustberg M et al. (2024) [2] conducted a pilot randomized controlled trial (RCT) with nanoemulsion curcumin (NEC) for alleviating aromatase inhibitor-induced arthropathy (AIIA) in postmenopausal women with ER-positive breast cancer. The study included postmenopausal women and, after 3 months, the NEC group showed detectable plasma curcumin levels. However, there was no significant difference in pain severity, interference, or symptom burden between the NEC and placebo groups. Elyasi S et al. (2022) [37] investigated the effects of topical henna and curcumin ointment on hand-foot syndrome (HFS) in 110 patients undergoing chemotherapy with capecitabine. The study found that the ointment did not significantly reduce the incidence of HFS compared to placebo. Both groups experienced an increase in HFS scores during treatment, but the curcumin ointment group showed a delay in HFS onset and slower progression of symptoms compared to the placebo group, particularly after the second and third chemotherapy courses. No significant differences were observed in the severity of HFS between the groups at the end of treatment, and both ointments were well-tolerated with no adverse effects reported.

Epigallocatechin-3-Gallate (EGCG): A potent antioxidant and anticancer catechin found in green tea. EGCG inhibits tumor growth, modulates estrogen receptors, and supports immune function. It also reduces inflammation and may enhance chemotherapy efficacy while minimizing toxicity [39]. These herbal extracts offer specific benefits in oncology, supporting the body’s response to chemotherapy and reducing treatment-related side effects.

In a randomized trial involving 165 patients with breast cancer undergoing postoperative radiotherapy, Epigallocatechin-3-Gallate (EGCG) supplementation was found to be effective in preventing radiation-induced dermatitis. The incidence of grade 2 or worse radiation-induced dermatitis was significantly lower in the EGCG group compared to the placebo group (50.5% vs. 72.2%; *p* = 0.008). Furthermore, EGCG supplementation delayed the onset of radiation-induced dermatitis symptoms [39].

#### 3.2.5. Green Tea Extract

Green tea extract, rich in polyphenols like catechins (primarily EGCG), offers antioxidant and anti-inflammatory benefits.

In a randomized, single-center cross-over trial involving 30 patients with breast cancer treated with tamoxifen, Braal et al. (2020) [40] evaluated the effects of green tea supplementation on endoxifen levels. The study found no significant differences in the geometric mean endoxifen AUC0-24h between periods of tamoxifen monotherapy and combined treatment with green tea capsules (1 g twice daily; containing 300 mg EGCG) (−0.4%; 95% CI −8.6 to 8.5%; *p* = 0.92). Additionally, no significant differences were observed in Cmax (−2.8%; −10.6 to 5.6%; *p* = 0.47) or Ctrough (1.2%; −7.3 to 10.5%; *p* = 0.77) between the two treatment periods. Importantly, the study reported no severe toxicity, highlighting that green tea supplementation did not alter tamoxifen’s efficacy, as indicated by stable endoxifen levels, although mild adverse events were more frequent with green tea. This study suggests that green tea does not interfere with tamoxifen’s effectiveness.

#### 3.2.6. Grape Seed Extract

Grape seed extract contains proanthocyanidins, powerful antioxidants that protect cells from oxidative stress and may inhibit cancer cell proliferation [41].

A double-blind, placebo-controlled phase II trial by Brooker et al. (2006) [41] involving 66 patients with radiation-induced breast induration tested the efficacy of grape seed proanthocyanidin extract (GSPE) over 12 months. Patients received 100 mg of GSPE or placebo three times daily. At the end of the study, a ≥50% reduction in induration surface area was observed in 29.5% of the GSPE group compared to 27% in the placebo group, which was not statistically significant. Furthermore, no significant differences were found between groups in tissue hardness, breast appearance, or self-assessments of pain and tenderness, indicating GSPE’s ineffectiveness. The results showed no statistically significant difference between the GSPE group and the placebo group in terms of reduction in induration surface area or other measures of breast health. This suggests that grape seed extract may not be effective in managing radiation-induced tissue changes.

#### 3.2.7. Phytoestrogens and Soy Isoflavones

Phytoestrogens, found in foods like flaxseed, soy, and red clover, mimic estrogen by binding to estrogen receptors. Soy isoflavones, such as genistein and daidzein, are phytoestrogens found in soy products. They can exert mild estrogenic or anti-estrogenic effects depending on hormone levels [10]. They are linked to lower cancer risk and improved survival rates, particularly in populations with high soy intake [42].

In a randomized placebo-controlled crossover trial, Nikander E et al. (2003) [43] assessed the effects of isoflavonoids on menopausal symptoms in 62 postmenopausal women with a history of breast cancer. The study found no significant differences between the phytoestrogen and placebo groups in reducing menopausal symptoms, as both groups showed similar reductions in the Kupperman index (15.5% vs. 14.7%, *p* non-significant). Quality of life metrics, such as working capacity and mood, also did not improve with isoflavonoids. In a 2-year randomized double-blind placebo-controlled trial, Lu LW et al. (2022) [10] investigated the effects of soy isoflavones on fibroglandular breast tissue (FGBT) in premenopausal women, finding statistically non-significant reductions in FGBT of 7.96% and 10.50% at the first and second MRIs, respectively (*p* = 0.071 and *p* = 0.080). After adjusting for BMI, the study observed mean decreases in FGBT of 5.3 cc, 12.1 cc, and 19.3 cc after 1.2, 2.2, and 3.3 years, respectively. Higher urinary excretion of isoflavone metabolites was linked to reduced FGBT. Additionally, Shike M et al. (2014) [42] conducted a study involving 140 women with invasive breast adenocarcinoma, which revealed that soy supplementation significantly increased plasma isoflavones but also altered gene expression in ways that could potentially be adverse. The analysis identified 21 altered genes, including significant changes in FANCC and UGT2A1, and a high-genistein signature characterized by the overexpression of 126 genes linked to cell cycle and proliferation pathways, including FGFR2. However, there were no significant changes in Ki67 or Cas3, indicating that soy intake might adversely impact gene expression in some breast cancer cases.

#### 3.2.8. Diosmin, Coumarin, and Arbutin

Diosmin, a flavonoid glycoside from citrus fruits, improves blood circulation and alleviates chronic venous insufficiency symptoms. In cancer patients, it helps manage circulatory issues, swelling, and fatigue, protecting against oxidative stress and reducing inflammation. Diosmin shows potential anticancer effects by modulating tumor growth pathways and may enhance treatment outcomes [44].

In a randomized controlled trial involving 48 patients with breast cancer-related lymphedema, Cacchio A et al. (2019) [44] evaluated the effectiveness of a product containing diosmin, coumarin, and arbutin combined with complex decongestive therapy versus a control treatment. After 3 months, the intervention group showed significant improvement in lymphedema severity, with 12.6% achieving minimal lymphedema compared to 31.8% in the control group, with no adverse events being reported.

#### 3.2.9. Decaffeinated Green Coffee Extract

Rich in chlorogenic acids, decaffeinated green coffee extract offers antioxidant and anti-inflammatory benefits, aiding in cancer care by regulating metabolism and reducing oxidative stress [45]. It inhibits cancer cell proliferation and induces apoptosis by modulating key cellular signaling pathways [45].

In a randomized clinical trial, Bahmannia M et al. (2022) [3] evaluated the effects of decaffeinated green coffee extract (DGCE) supplementation on various health metrics in 44 breast cancer survivors with obesity. Participants received either 800 mg of DGCE or a placebo daily for 12 weeks. The study found no significant differences between the DGCE and placebo groups in terms of changes in anthropometric indices (e.g., body weight, BMI), blood glucose levels, and serum concentrations of leptin, adiponectin, and neuropeptide Y (NPY). Therefore, DGCE supplementation did not significantly impact these health outcomes in breast cancer survivors with obesity.

### 3.3. Risk of Bias and Study Quality Assessment

The overall quality of the included trials varied, with most demonstrating a low risk of bias, while several had some concerns, particularly due to open-label designs and missing data. Studies such as those by Tirgar et al. [1], Erfanian et al. [35], Dastmardi et al. [22], McGuinness et al. [7], Parsowith et al. [33], Lustberg et al. [2], and Mukhopadhyay et al. [6] employed robust randomization methods, placebo-controlled designs, and blinding strategies that minimized performance and detection bias. These trials consistently reported comprehensive outcome data, with low rates of attrition and well-documented statistical analyses, reinforcing their reliability.

By contrast, several trials, including those by Arsic et al. [9], Lu et al. [10], Bahmannia et al. [3], Ostadrahimi et al. [32], and Ghoreishi et al. [28], were flagged for concerns primarily due to open-label designs or notable missing data that could introduce bias. Arsic et al. [9] did not blind participants or outcome assessors, increasing the potential for performance and detection bias. Similarly, Lu et al. [10] reported a dropout of 12 out of 88 participants, potentially leading to attrition bias, while Ghoreishi et al. [28] also had missing outcome data with 12 participants lost to follow-up, raising concerns about selective results. In the trial by Ostadrahimi et al. [32] it was unclear whether the intervention and placebo were identical in appearance, creating a functional unblinding risk.

Despite these limitations, most studies demonstrated strong transparency in reporting, with clear pre-specified outcomes and comprehensive statistical analyses.

## 4. Discussion

This systematic review examined the effects of various dietary supplements on patients with breast cancer, emphasizing their impact on clinical outcomes, side effects, and overall quality of life. It is important to note that the use of dietary supplements in cancer care should be considered in the context of established guidelines and potential interactions with conventional treatments. Organizations such as the World Health Organization (WHO) and the American Institute for Cancer Research (AICR) generally recommend that cancer patients obtain necessary nutrients through a balanced diet rather than relying solely on supplements (https://www.aicr.org/cancer-prevention/healthy-eating/supplements-nutrients/, accessed on 20 February 2025). However, they acknowledge that supplements may be beneficial in specific situations, such as when nutritional deficiencies exist or when there is evidence of therapeutic benefit. Our findings on the potential benefits of Omega-3 fatty acids and beta glucan are consistent with these guidelines, as these supplements appear to improve quality of life and support immune function in breast cancer patients. However, the inconsistent results observed with Vitamin D and other supplements emphasize the need for careful consideration and personalized approaches. Furthermore, it is crucial to be aware of potential interactions between dietary supplements and medications commonly used in breast cancer treatment. For example, some supplements may interfere with the metabolism of chemotherapy drugs, potentially affecting their efficacy or increasing the risk of side effects. Similarly, certain supplements may interact with hormonal therapies, such as tamoxifen, potentially altering their effectiveness. Healthcare providers should carefully evaluate the potential for drug–nutrient interactions before recommending any dietary supplements to breast cancer patients.

This review acknowledges several limitations, including the heterogeneity of study designs, sample sizes, and intervention dosages, which may impact the generalizability of the results. The variability in outcome measures and the lack of standardization in reporting further complicate the comparison of results across studies. Additionally, the lack of long-term follow-up in many studies limits the understanding of the sustained effects of these supplements. Addressing these limitations in future research is crucial to provide more conclusive evidence on the efficacy and safety of dietary supplements in breast cancer care. Compared to previous systematic reviews and meta-analyses, this review offers a comprehensive synthesis of recent RCTs, highlighting both the potential benefits and limitations of dietary supplements in breast cancer care. Previous reviews have similarly noted the variability in supplement efficacy, underscoring the importance of personalized approaches and further research. The inconsistency in findings regarding Vitamin D supplementation aligns with earlier research showing mixed outcomes depending on dosage, patient demographics, and concurrent therapies.

The current landscape of dietary supplement research in breast cancer care highlights significant gaps and opportunities for innovation. Dietary supplements have shown promise in improving quality of life, managing treatment-related side effects, and potentially influencing clinical outcomes [46]. However, the evidence base remains inconsistent, with studies differing in supplement types, dosages, populations, and outcome measures, making it challenging to draw generalized conclusions. The variability in study designs, particularly the lack of standardization in formulations and methodologies, hampers comparability across trials and limits the reliability of pooled data. For instance, differences in bioavailability of curcumin formulations or the variability in Omega-3 fatty acid content complicate the evaluation of their clinical utility [47]. While randomized controlled trials (RCTs) are considered the gold standard for evaluating interventions, they often fall short in capturing the nuanced benefits and challenges of dietary supplements, particularly in complex diseases like breast cancer. RCTs are costly, time-intensive, and constrained by artificial study conditions that may not reflect routine clinical practice, leading to results that are sometimes difficult to translate into real-world applications.

Many studies focus on immediate or short-term effects, such as alleviation of chemotherapy side effects, but fail to assess long-term outcomes like disease-free survival or recurrence rates [48]. This focus on surrogate biomarkers—although valuable for mechanistic insights—may not always correlate with clinically meaningful endpoints, limiting their relevance for patient care. Moreover, the underlying biological mechanisms through which supplements exert their effects remain inadequately understood [49]. Supplements such as Vitamin D, Omega-3 fatty acids, and curcumin [50] are believed to act through pathways involving inflammation, oxidative stress, and immune modulation, but the precise molecular targets and downstream effects remain speculative. A deeper understanding of these mechanisms is essential to identify which patient subgroups might benefit most from specific interventions. For instance, the potential differential effects of Vitamin D supplementation based on baseline serum levels or genetic polymorphisms in vitamin metabolism pathways remain underexplored, despite their implications for personalized medicine [51].

Given these limitations, innovative strategies are needed to advance research and practice in dietary supplements. Pragmatic trials, which reflect real-world clinical settings, offer a promising alternative to traditional RCTs. Unlike tightly controlled trials, pragmatic trials allow for greater flexibility in participant selection, dosing regimens, and concurrent therapies, capturing data that are more representative of routine clinical practice [52]. For example, a pragmatic trial could evaluate the impact of Omega-3 supplementation on quality of life in patients with breast cancer across diverse treatment settings, using patient-reported outcomes and adherence rates as primary endpoints. Similarly, observational studies, while traditionally viewed as less rigorous than RCTs, provide valuable opportunities to study supplements in real-world contexts. Large-scale cohort studies and registries can track supplement use over time, generating hypotheses about their potential benefits and risks. Advances in data science and machine learning enhance the potential of observational research by enabling the analysis of complex datasets, such as those derived from electronic health records or wearable devices [53].

A critical issue in supplement research is the regulatory landscape, which allows for supplements to be marketed without formal clinical trials, provided they meet basic safety standards. While this regulatory flexibility facilitates market entry, it also discourages rigorous research, as manufacturers have little incentive to invest in costly studies. Addressing this issue requires policies that incentivize evidence generation, such as tax credits for clinical research, public–private partnerships, or conditional marketing approvals tied to post-market studies. These measures could help balance the accessibility of supplements with the need for robust evidence to support their use. Bridging the gap between research and clinical practice is essential for maximizing the benefits of dietary supplements. This requires the integration of supplements into comprehensive care models that prioritize patient-centered outcomes. Collaboration among oncologists, nutritionists, and integrative medicine specialists is crucial for developing evidence-based guidelines. Educational initiatives targeting healthcare providers and patients can promote informed decision-making, ensuring that supplements are used appropriately and effectively. Digital health tools, such as mobile apps and wearable devices, further enhance research and practice by facilitating real-time data collection and monitoring. For instance, an app could track patients’ supplement use, side effects, and quality of life, providing valuable data for both clinical care and research. Wearable devices could measure objective outcomes, such as physical activity or sleep quality, offering additional insights into the effects of supplements.

A further crucial limitation of the current evidence base, and consequently of this review, is the predominant focus on short-term outcomes in many of the included studies. While improvements in quality of life, treatment adherence, and management of acute side effects (such as chemotherapy-induced nausea or fatigue) are undoubtedly important for patients undergoing breast cancer treatment, the ultimate goals of cancer therapy are to improve long-term survival and reduce the risk of recurrence. Many of the reviewed RCTs had follow-up periods of only weeks or months, often coinciding with the duration of active treatment (e.g., chemotherapy or radiation therapy). This short-term perspective makes it impossible to determine whether any observed benefits of supplementation translate into improved disease-free or overall survival. It is biologically plausible that some supplements, particularly those with anti-inflammatory, antioxidant, or immunomodulatory properties (such as omega-3 fatty acids, Vitamin D, or curcumin), could influence long-term outcomes. For example, chronic inflammation is implicated in cancer progression and metastasis, and supplements that effectively reduce inflammation might, in theory, reduce the risk of recurrence. Similarly, supplements that enhance immune function could potentially improve the body’s ability to eliminate residual cancer cells. However, these remain hypotheses that require rigorous testing in long-term studies. Therefore, we strongly recommend that future research in this area prioritize long-term follow-up, ideally extending for several years after the completion of primary cancer treatment. Studies should specifically assess disease-free survival, overall survival, and the incidence of late-onset toxicities as primary or secondary outcomes. Furthermore, future trials should consider incorporating biomarker analyses (e.g., measuring inflammatory markers, circulating tumor cells, or immune cell profiles) to provide mechanistic insights into any observed long-term effects. Cohort studies, while potentially subject to confounding, could also provide valuable long-term data on supplement use and cancer outcomes, particularly if they leverage existing large datasets and biobanks. Only with such long-term data can we truly understand the potential role of dietary supplements in improving the prognosis of breast cancer patients.

Real-world evidence initiatives and innovative trial designs represent the future of dietary supplement research, offering a path to overcome the limitations of traditional methodologies. By reflecting real-world clinical practices and focusing on patient-centered outcomes, these approaches can provide meaningful insights into the role of supplements in breast cancer care. Policies that incentivize research, coupled with collaborative efforts across disciplines and regions, are essential to advancing the field and ensuring that dietary supplements are used safely and effectively. This shift toward context-specific, patient-centered research represents a promising new frontier in the integration of complementary therapies into oncology practice.

## 5. Conclusions

This systematic review synthesized evidence on dietary supplements in breast cancer care, not to establish formal clinical guidelines but to inform potential clinical applications. The current evidence base remains limited, heterogeneous, and often focused on short-term outcomes, necessitating cautious interpretation. As such, the following insights should be considered as preliminary suggestions rather than definitive recommendations. Any decision regarding supplement use must involve shared decision-making between patients and healthcare providers, tailored to individual clinical needs, preferences, and risk–benefit considerations.

For Vitamin D, while evidence remains mixed, clinicians might consider evaluating and addressing deficiencies (serum 25(OH)D < 30 ng/mL) primarily to support bone health. Omega-3 fatty acids (EPA/DHA) at doses of 1–4 g per day (typically in a 2:1 EPA:DHA ratio) could be explored for managing chemotherapy-induced xerostomia or neuropathy, though potential bleeding risks, particularly with anticoagulants or antiplatelets, must be considered. Beta-glucan (e.g., 10 mg daily, source unspecified) may offer immune support for patients undergoing chemotherapy and at high risk of infection, though data remain preliminary. Curcumin, particularly in bioavailable formulations, shows promise for alleviating specific treatment-related side effects but requires further validation. Silymarin (standardized extracts, 70–80% silymarin) may serve as a hepatoprotective agent during chemotherapy, though its impact on renal function remains unclear. Epigallocatechin gallate (EGCG) has insufficient evidence to support use alongside tamoxifen, and grape seed extract has yet to be substantiated for reducing radiation-induced breast induration. Other supplements, including melatonin, creatine, phytoestrogens, soy isoflavones, diosmin, coumarin, arbutin, and decaffeinated green coffee extract, lack sufficient or conclusive evidence to justify clinical recommendations. Crucially, dietary supplements should never replace conventional cancer treatments, and their role must be carefully weighed against established therapies. Future research should prioritize large-scale, long-term randomized controlled trials (RCTs) to determine optimal dosages, formulations, timing, and long-term effects on survival and recurrence.

In summary, this review encompassed 45 RCTs examining dietary supplements in breast cancer patients. Despite the study heterogeneity limiting broad conclusions, several notable findings emerged. Omega-3 fatty acids and beta-glucan demonstrated some degree of benefits, particularly in enhancing quality of life, mitigating chemotherapy-induced side effects, and supporting immune function. These findings suggest potential roles as adjunctive therapies warranting further exploration in larger, long-term studies. Vitamin D, silymarin, curcumin, and various herbal extracts produced more variable results, reinforcing the need for personalized approaches to supplementation based on patient characteristics, tumor biology, and treatment regimens. For example, while Vitamin D exhibited some anti-inflammatory effects, its impact on tumor progression remains inconclusive. Similarly, silymarin showed promise for hepatic protection during chemotherapy, but further research is needed to assess its effects on renal function.

Future investigations should focus on rigorous, well-designed RCTs with extended follow-up periods to validate these preliminary findings and elucidate underlying mechanisms. Understanding potential synergies between supplements and standard therapies, as well as developing personalized supplementation strategies, will be critical in optimizing their clinical utility. Such an approach could enhance treatment outcomes and quality of life for breast cancer patients, fostering a more integrative and evidence-based model of care.

## Data Availability

The data presented in this study are available upon request from the corresponding author due to privacy safeguards.

## Supplementary Materials

The following supporting information can be downloaded at: https://www.mdpi.com/article/10.3390/nu17060981/s1, Table S1: Synthesis of included trials; Table S2: Summary Table for each supplements.
