# Supplementary material for: Impact of Dietary Supplements on Clinical Outcomes and Quality of Life in Patients with Breast Cancer: A Systematic Review
Source: Nutrients. 2025 Mar 11;17(6):981. doi: 10.3390/nu17060981 (PMC11945011; doi:10.3390/nu17060981)
Supplement: Supplementary file 1 [file nutrients-17-00981-s001.zip › nutrients-3502734-supplementary.pdf]

Table S1: Synthesis of included trials

Study ID (Author, Year)	Supplement Category	Participants	Intervention	Control	Outcomes	Risk of Bias	Key Findings
39266591 Tirgar A et al (2024)	Vitamin D and Synbiotics	Breast cancer patients	Vitamin D + synbiotics	Placebo	Cytokine profile, treatment response	Low	Synergistic effect of Vitamin D and synbiotics improved cytokine profile and treatment response in breast cancer patients.
39232773 Erfanian SS et al (2024)	Silymarin (Herbal)	Cancer patients receiving chemotherapy	Silymarin	Placebo	Hepatorenal protection	Low	Silymarin supplementation protected against chemotherapy-induced hepatorenal toxicity.
38925607 Dastmardi Z et al (2024)	Vitamin D	Breast cancer patients undergoing chemotherapy	Vitamin D	Placebo	Ovarian reserve, Anti-Mullerian hormone	Low	Vitamin D preserved ovarian reserve and maintained Anti-Mullerian hormone levels during chemotherapy.
38814817 McGuinness JE et al (2024)	Vitamin D	Breast cancer patients	Vitamin D	Placebo	Mammographic evaluation using deep learning	Low	Vitamin D supplementation influenced mammographic density in breast cancer patients, as analyzed through deep learning models.
38613014 Parsowith EJ et al (2024)	Creatine	Breast cancer survivors	Short-term creatine supplementation	Placebo	Muscular performance	Low	Short-term creatine supplementation improved muscular performance in breast cancer survivors.
38280052 Lustberg M et al (2024)	Curcumin (Nanoemulsion)	Women with aromatase inhibitor-induced arthropathy	Curcumin (nanoemulsion)	Placebo	Arthropathy symptoms	Low	Curcumin nanoemulsion alleviated arthropathy symptoms in breast cancer patients on aromatase inhibitors.
37752516 Khazaei Y et al (2023)	Synbiotics	Women with breast cancer undergoing chemotherapy	Synbiotics	Placebo	Chemotherapy-induced side effects	Low	Synbiotic supplementation reduced chemotherapy-induced gastrointestinal side effects in breast cancer patients.
35331230 Naderi M et al (2022)	Vitamin D + Yoga	Breast cancer survivors	Yoga and vitamin D supplementation	None	Serum cytokine and gene expression	Low	Combination of yoga and vitamin D showed favorable effects on cytokine levels and gene expression in breast cancer survivors.
37699115 Mukhopadhyay ND et al 2024	Melatonin	Breast cancer patients receiving radiotherapy	Melatonin supplementation	Placebo	Cancer-related fatigue reduction, quality of life	Low	Melatonin significantly reduced cancer-related fatigue and improved quality of life in breast cancer patients.
37081029 Arsic A et al (2023)	Fish Oil and Evening Primrose Oil	Breast cancer patients undergoing chemotherapy	Combination of fish oil and evening primrose oil	Placebo	Anti-inflammatory effects, quality of life, treatment-related side effects	Some concerns	Significant anti-inflammatory effects observed in breast cancer patients undergoing chemotherapy.
36513449 Lu W et al (2022)	Soy Isoflavones	Premenopausal women with breast cancer	Soy isoflavone supplementation	Placebo	Reduction in fibroglandular breast tissue, breast cancer progression markers	Some concerns	Soy isoflavones significantly decreased fibroglandular breast tissue measured by MRI in premenopausal women.
36134465 Bahmannia M et al (2022)	Decaffeinated Green Coffee Extract	Breast cancer survivors	Decaffeinated green coffee extract supplementation	Placebo	Anthropometric indices, blood glucose, leptin, adiponectin, neuropeptide Y (NPY) levels	Some concerns	Improvements in anthropometric indices and regulation of blood glucose and leptin in breast cancer survivors.
35747962 Talakesh T et al (2022)	Nano-Curcumin	Breast cancer patients undergoing radiotherapy	Nano-curcumin supplementation	Placebo	Skin reaction, radiotherapy-induced dermatitis	Low	Nano-curcumin significantly reduced radiotherapy-induced skin reactions.

35648426 Zhao H et al (2022)	Epigallocatechin-3-Gallate	165 Breast cancer patients receiving postoperative radiotherapy	Epigallocatechin-3-Gallate supplementation	Placebo	Prevention of radiodermatitis, treatment-related side effects	Low	Epigallocatechin-3-Gallate was effective in preventing dermatitis in patients receiving postoperative radiotherapy.
35320555 Elyasi S et al (2022)	Curcumin + Henna	Breast cancer patients on capecitabine	Topical curcumin + henna ointment	Placebo	Prevention of hand-foot syndrome	Low	Curcumin and henna ointment reduced incidence and severity of capecitabine-induced hand-foot syndrome.
34085881 Saneei Totmaj A et al (2022)	Synbiotics + Diet	Breast cancer survivors with lymphedema	Synbiotics and low-calorie diet	Placebo	Anti-inflammatory markers in lymphedema	Low	Synbiotic supplementation and diet improved serum anti-inflammatory markers and symptoms of lymphedema.
33861657 Moezian GSA et al (2022)	Silymarin	Breast cancer patients undergoing AC-T protocol	Oral silymarin	Placebo	Hepatotoxicity from chemotherapy	Low	Silymarin was effective in reducing chemotherapy-induced hepatotoxicity in breast cancer patients.
33051045 (Bracone, 2020)	Anthocyanin	Breast cancer patients receiving radiotherapy	Anthocyanin supplementation	Placebo	Reduction in skin toxicity following radiotherapy	Some concerns	Anthocyanin supplementation showed beneficial effects in reducing radiotherapy-induced skin toxicity.
32898787 (Vafa, 2020)	Calorie restriction and synbiotics	Breast cancer patients with lymphedema	Calorie restriction and synbiotic supplementation	Standard care	Improvement in quality of life and reduction of edema	Some concerns	Combination of calorie restriction and synbiotics improved quality of life and reduced edema in lymphedema.
32803636 (Braal, 2020)	Green tea	Breast cancer patients on tamoxifen	Green tea supplementation	Placebo	Endoxifen steady-state concentration, tamoxifen efficacy	Low	Green tea consumption did not affect endoxifen steady-state concentrations in tamoxifen-treated patients.
31901710 (Shahvegharasl, 2020)	Cholecalciferol (Vitamin D3)	Breast cancer patients on tamoxifen	Cholecalciferol supplementation	Placebo	Serum angiogenic biomarkers	Some concerns	Vitamin D3 supplementation modulated serum angiogenic biomarkers in tamoxifen-treated breast cancer patients.
31350967 (Mohseni, 2019)	Vitamin D3	Breast cancer patients	Vitamin D3 supplementation	Placebo	Inflammatory markers, total antioxidant capacity	Some concerns	Vitamin D3 supplementation modulated inflammatory markers and improved antioxidant capacity.
31218477 (Niravath, 2019)	Vitamin D3	Breast cancer patients on aromatase inhibitors	High-dose vs standard-dose Vitamin D3	Standard-dose	Prevention of aromatase inhibitor-induced arthralgia	Low	High-dose Vitamin D3 was more effective in preventing aromatase inhibitor-induced arthralgia.
31192682 (de la Rosa Oliva, 2019)	Omega-3 fatty acids	Breast cancer patients receiving neoadjuvant chemotherapy	Omega-3 fatty acid supplementation	Placebo	Chemotherapy-induced toxicity, quality of life	Some concerns	Omega-3 supplementation reduced chemotherapy-induced toxicity and improved quality of life.
30912414 (Darwito, 2019)	Omega-3 fatty acids	Breast cancer patients receiving neoadjuvant chemotherapy	Omega-3 supplementation	Placebo	Ki-67 and VEGF expression, clinical outcomes	Some concerns	Omega-3 supplementation improved clinical outcomes and modulated Ki-67 and VEGF expression levels.
30479044 Karbasforooshan H et al (2019)	Topical silymarin	Breast cancer patients	Topical silymarin	Placebo	Prevention of radiodermatitis	Low	Silymarin showed efficacy in reducing acute radiodermatitis severity in breast cancer patients undergoing radiation therapy.
30343411 Cacchio A et al (2019)	Diosmin, coumarin, arbutin (Linfadren®)	Breast cancer survivors with lymphedema	Diosmin, coumarin, and arbutin (Linfadren®)	Placebo	Lymphedema symptoms and management	Low	Linfadren® improved symptoms of lymphedema in breast cancer patients.
29361042 Hershman DL et al (2018)	Acetyl-L-carnitine	Breast cancer patients un-	Acetyl-L-carnitine	Placebo	Taxane-induced neuropathy	Low	No significant benefit was found in preventing taxane-induced neuropathy with acetyl-L-carnitine.

		dergoing taxane chemotherapy					
29101597 Lustberg MB et al (2018)	Omega-3 fatty acids	Breast cancer patients on aromatase inhibitors	Omega-3 fatty acids	Placebo	Musculoskeletal pain from aromatase inhibitors	Low	Omega-3 fatty acids reduced musculoskeletal pain associated with aromatase inhibitors in breast cancer patients.
25190728 Shike M, et al (2014)	Soy Supplementation	Breast cancer patients	Soy supplementation	Placebo	Gene expression changes related to breast cancer	Low	Soy supplementation affected gene expression but its clinical relevance remains unclear.
25081694 Ostadrahimi A et al (2014)	Beta Glucan	Women with breast cancer undergoing chemotherapy	Beta glucan supplementation	Placebo	Changes in white blood cell counts and serum levels of IL-4 and IL-12	Some concerns	Beta glucan had a positive effect on immune function during chemotherapy.
24994839 Pouchieu C et al (2014)	Antioxidants and Meat Intake	Breast cancer survivors	Antioxidant supplementation (SU.VI.MAX study)	No antioxidant supplementation	Breast cancer risk related to red and processed meat intake	Low	Antioxidant supplementation may reduce the risk of breast cancer in women consuming higher red and processed meat.
24274259 Hutchins-Wiese HL (2014)	Omega-3 Fatty Acids	Postmenopausal breast cancer survivors on aromatase inhibitors	High-dose eicosapentaenoic acid (EPA) and docosahexaenoic acid (DHA) supplementation	Placebo	Bone resorption rates in breast cancer survivors receiving aromatase inhibitors	Low	High-dose EPA and DHA supplementation reduced bone resorption in postmenopausal breast cancer survivors on aromatase inhibitors.
23833361 (Valadares F, 2013)	Herbal Extract	30 women with breast cancer	Agaricus sylvaticus supplementation	Placebo	Nutritional status, adverse events	Low	Improved nutritional status, reduced side effects of chemotherapy
23745991 (Ryan JL, 2013)	Herbal Extract	30 women with breast cancer	Curcumin for radiation dermatitis	Placebo	Radiation dermatitis severity	Low	Significant reduction in dermatitis severity
23733756 (Hershman DL, 2013)	Single Well-Defined Substance	100 women with breast cancer	Acetyl-L-carnitine for neuropathy prevention	Placebo	Neuropathy incidence	Some concerns	Reduced incidence of neuropathy
22894640 (Ghoreishi Z, 2012)	Omega-3 Fatty Acids	80 women with breast cancer	Omega-3 fatty acids	Placebo	Peripheral neuropathy, quality of life	Some concerns	Omega-3 fatty acids reduced neuropathy symptoms
22370698 (Schernhammer ES, 2012)	Single Well-Defined Substance	140 women with breast cancer	Oral melatonin supplementation	Placebo	Breast cancer biomarkers	Some concerns	Melatonin supplementation positively affected biomarkers
18853250 (Rohan TE, 2009)	Single Well-Defined Substance	120 women with benign proliferative breast disease	Calcium plus vitamin D supplementation	Placebo	Benign proliferative breast disease risk	Low	Calcium and vitamin D did not significantly affect risk
17575230 (Llombart-Cussac A, 2007)	Single Well-Defined Substance	500 women with advanced breast cancer	Pemetrexed chemotherapy	Placebo	Tumor response, overall survival	Some concerns	Significant improvement in tumor response
19540105 Magnusson M et al. (2009)	Pentoxifylline + Vitamin E	Women with breast cancer	Pentoxifylline + Vitamin E	Placebo	Radiation-induced side effects	Low	Showed potential in preventing radiation side effects.
17762445 de Souza Fêde AB et al. (2007)	Multivitamins	Cancer patients	Multivitamin supplementation	Placebo	Radiation therapy-related fatigue	Low	No improvement in fatigue observed.
16546280 Brooker S et al. (2006)	Grape seed proanthocyanidin extract	Patients with breast cancer	Grape seed extract	Placebo	Radiation-induced breast induration	Low	Potential benefits for radiation-induced effects.
12798527 Nikander E et al. (2003)	Phytoestrogens	Breast cancer patients	Phytoestrogen supplementation	Placebo	Menopausal symptoms assessment	Low	Positive effects on menopausal symptoms observed.
9454908 Heys SD et al. (1998)	L-arginine supplementation	Breast cancer patients	Dietary supplementation with L-arginine	Control group	Chemotherapy response assessment	Low	Positive response to chemotherapy noted.

Table S2: Summary Table for each supplement

Supplement	Study Reference	Sample Size	Population	Intervention	Control	Main Findings	Conclusion
**Vitamin D and Minerals**	Tirgar et al., 2024	100	Breast cancer patients	Vitamin D + Synbiotics	Placebo	Stabilization of IL-10; no significant impact on tumor markers	Anti-inflammatory effect, not tumor control
	Dastmardi et al., 2024	120	Chemotherapy patients	Vitamin D	Placebo	Non-significant trend towards improved AMH levels	Limited effect on ovarian reserve
	McGuinness et al., 2024	80	Breast cancer survivors	Vitamin D	Placebo	No significant changes in mammographic density risk indicators	Inconclusive due to limited sample size
	Naderi et al., 2022	90	Breast cancer survivors	High-dose Vitamin D + Yoga	Placebo	Enhanced IL-10 levels in combination group	Lifestyle factors could amplify Vitamin D effects
	Shahvegharasl, 2020	110	Tamoxifen-treated patients	Vitamin D3	Placebo	No effect on angiogenic biomarkers	Vitamin D3 not effective for angiogenic biomarkers
	Mohseni, 2019	95	Breast cancer patients	Vitamin D3	Placebo	Improved antioxidant capacity; no significant impact on inflammatory markers	Antioxidant benefits without significant inflammatory impact
	Niravath et al., 2019	105	AI-induced arthralgia	High-dose Vitamin D3	Placebo	Raised serum levels; no reduction in arthralgia	High-dose Vitamin D3 not effective for arthralgia
**Omega-3 Fatty Acids**	De la Rosa Oliva et al., 2019	150	Chemotherapy patients	Omega-3	Placebo	Improved quality of life and reduced xerostomia	Omega-3 beneficial for QoL and xerostomia
	Darwito et al., 2019	160	Chemotherapy patients	Omega-3	Placebo	Enhanced progression-free and overall survival; reduced Ki-67 and VEGF levels	Omega-3 may aid tumor modulation
	Lustberg et al., 2018	140	AI-treated patients	Omega-3	Placebo	Maintained quality of life; no significant reduction in pain	Omega-3 maintains QoL during AI therapy
	Hutchins-Wiese et al., 2014	130	Postmenopausal patients	EPA and DHA	Placebo	Significant reduction in bone resorption rates	Omega-3 beneficial for bone health
	Ghoreishi et al., 2012	120	Chemotherapy patients	Omega-3	Placebo	Reduced chemotherapy-induced neuropathy; preserved nerve conduction	Omega-3 reduces neuropathy in chemotherapy

**Amino Acids**	SWOG S0715	200	Taxane-treated patients	Acetyl-L-carnitine	Placebo	Worsened neuropathy symptoms in some patients	ALC not beneficial for neuropathy management
	L-Arginine trial	150	Breast cancer patients	L-arginine	Placebo	Enhanced chemotherapy response in patients with smaller tumors	L-arginine shows selective benefit in treatment efficacy
**Other Supplements**	Parsowith et al., 2024	120	Breast cancer survivors	Creatine	Placebo	Improved muscular performance over time; no clear advantage over placebo	Creatine improves muscular performance, mixed outcomes
	Mukhopadhyay et al., 2024	100	Radiotherapy patients	Melatonin	Placebo	No significant improvement in fatigue; well tolerated	Melatonin well tolerated, no significant impact on fatigue
	Ostadrahimi et al., 2014	90	Chemotherapy patients	Beta glucan	Placebo	Reduced drop in white blood cells; increased IL-4 and IL-12 levels	Beta glucan helps mitigate immunosuppression
**Well-Defined Substances**	Erfanian et al., 2024	100	Chemotherapy patients	Silymarin	Placebo	Improved liver function markers; no significant change in renal markers	Silymarin offers liver protection; renal benefit inconclusive
	Moezian et al., 2022	56	AC-T chemotherapy patients	Silymarin	Placebo	Improved antioxidant capacity	Silymarin enhances antioxidant capacity
	Lustberg et al., 2024	150	AI-treated patients	Nanoemulsion curcumin	Placebo	No significant reduction in arthralgia	Nanoemulsion curcumin well tolerated, not effective for arthralgia
	Elyasi et al., 2022	160	Capecitabine-treated patients	Curcumin-henna ointment	Placebo	Delayed onset of HFS; no adverse effects	Curcumin-henna ointment delays HFS onset
**Herbal Extracts**	Zhao H et al., 2022	165	Radiotherapy patients	EGCG (Green Tea Extract)	Placebo	Reduced incidence and severity of RID	EGCG reduces RID severity and incidence
	Braal et al., 2020	14	Tamoxifen-treated patients	Green Tea Extract	Placebo	No alteration in tamoxifen efficacy; mild adverse events more frequent	Green tea does not interfere with tamoxifen effectiveness
	Brooker et al., 2006	66	Radiotherapy patients	Grape Seed Extract	Placebo	No significant reduction in breast induration	Grape seed extract not effective for ra-

							diation-induced tissue changes
	Nikander et al., 2003	62	Breast cancer survivors	Phytoestrogens	Placebo	No significant impact on menopausal symptoms	Phytoestrogens not effective for menopausal symptoms
	Lu et al., 2022	120	Breast cancer patients	Soy Isoflavones	Placebo	Potential reduction in breast density markers; clinical significance unclear	Soy isoflavones show potential, clinical relevance needs further exploration
	Shike et al., 2014	150	Breast cancer patients	Soy supplementation	Placebo	Changes in gene expression; no difference in proliferation markers	Soy influences molecular pathways, clinical relevance needs more research
